# Supplementary material for: Multimodal Tracking of Hematopoietic Stem Cells from Young and Old Mice Labeled with Magnetic–Fluorescent Nanoparticles and Their Grafting by Bioluminescence in a Bone Marrow Transplant Model
Source: Biomedicines. 2021 Jun 29;9(7):752. doi: 10.3390/biomedicines9070752 (PMC8301491; doi:10.3390/biomedicines9070752)
Supplement: Supplementary file 1 [file biomedicines-09-00752-s001.zip › Supplementary Materials.pdf]

Supplementary Materials

Table S1. ANOVA of the BLI intensity of BM-MNC from young and old mice labeled with different concentrations of $SPION_{NIRF\text{-}Rh}$.

Variation source	Sum of squares	Degrees of freedom	Mean square	F	p
Applications	$1.32x10^{14}$	7	$1.88X10^{13}$	24.10	< 0.001
Residual	$2.50x10^{13}$	32	$7.81x10^{11}$		

$SPION_{NIRF\text{-}Rh}$: multimodal nanoparticles; BM-MNC: bone marrow mononuclear cells; BLI: Bioluminescence.

Table S2. Post Hoc test of the BLI intensity of BM-MNC from young and old mice labeled with different concentrations of $SPION_{NIRF\text{-}Rh}$.

Groups		Mean difference	95% CI		P
			Lower Bound	Upper Bound	
ctrl_young	ctrl_old	$8.66x10^5$	$5.59x10^5$	1.55	1
	c10_young	$1.77x10^6$	$5.59x10^5$	3.17	0.094
	c10_old	$2.28x10^6$	$5.59x10^5$	4.09	0.008
	c30_young	$3.48x10^6$	$5.59x10^5$	6.22	< 0.001
	c30_old	$4.13x10^6$	$5.59x10^5$	7.39	< 0.001
	c50_young	$5.21x10^6$	$5.59x10^5$	9.32	< 0.001
	c50_old	$5.13x10^6$	$5.59x10^5$	9.18	< 0.001
ctrl_old	c10_young	$9.06x10^5$	$5.59x10^5$	1.62	1
	c10_old	$1.42x10^6$	$5.59x10^5$	2.54	0.454
	c30_young	$2.61x10^6$	$5.59x10^5$	4.68	0.001
	c30_old	$3.27x10^6$	$5.59x10^5$	5.84	< 0.001
	c50_young	$4.34x10^6$	$5.59x10^5$	7.77	< 0.001
	c50_old	$4.27x10^6$	$5.59x10^5$	7.63	< 0.001
c10_young	c10_old	$5.12x10^5$	$5.59x10^5$	0.92	1
	c30_young	$1.70x10^6$	$5.59x10^5$	3.05	0.127
	c30_old	$2.36x10^6$	$5.59x10^5$	4.30	0.005
	c50_young	$3.44x10^6$	$5.59x10^5$	6.15	< 0.001
	c50_old	$3.36x10^6$	$5.59x10^5$	6.01	< 0.001
c10_old	c30_young	$1.19x10^6$	$5.59x10^5$	2.14	1
	c30_old	$1.85x10^6$	$5.59x10^5$	3.30	0.066
	c50_jovens	$2.92x10^6$	$5.59x10^5$	5.23	< 0.001
	c50_old	$2.85x10^6$	$5.59x10^5$	5.09	< 0.001
c30_young	c30_old	$6.52x10^5$	$5.59x10^5$	1.17	1
	c50_young	$1.73x10^6$	$5.59x10^5$	3.10	0.114
	c50_old	$1.65x10^6$	$5.59x10^5$	2.96	0.162
c30_old	c50_young	$1.08x10^6$	$5.59x10^5$	1.93	1
	c50_old	$1.00x10^6$	$5.59x10^5$	1.80	1
c50_young	c50_old	-7.7610^5	$5.59x10^5$	-0.14	1

$SPION_{NIRF\text{-}Rh}$: multimodal nanoparticles; BM-MNC: bone marrow mononuclear cells; BLI: Bioluminescence; ctrl: Control; c10: concentration of 10 µg Fe/mL of $SPION_{NIRF\text{-}Rh;}$ c30: concentration of 30 µg Fe/mL of $SPION_{NIRF\text{-}Rh;}$ c50: concentration of 50 µg Fe/mL of $SPION_{NIRF\text{-}Rh}$; CI: confidence interval.

Table S3. ANOVA test of the NIRF intensity of BM-MNC from young and old animals labeled with 10, 30, and 50 μg Fe/mL of $SPION_{NIRF-Rh}$.

Variation source	Sum of squares	Degrees of freedom	Mean square	F	p
Applications	$3.82x10^{17}$	5	$7.64X10^{16}$	132.20	< 0.001
Residual	$1.39x10^{16}$	24	$5.78x10^{14}$		

$SPION_{NIRF-Rh}$: multimodal nanoparticles; BM-MNC: bone marrow mononuclear cells; NIRF: Near-infrared fluorescence.

Table S4. Post Hoc test of the NIRF intensity of BM-MNC from young and old animals labeled with 10, 30, and 50 μg Fe/mL of $SPION_{NIRF-Rh}$.

Group		Mean difference	95% CI		95% CI
			Lower Bound	Upper Bound	
c10_young	c10_old	$4.54x10^{7}$	$1.52x10^{7}$	2.97	0.096
	c30_young	$-1.30x10^{8}$	$1.52x10^{7}$	-8.55	< 0.001
	c30_old	$-1.21x10^{8}$	$1.52x10^{7}$	-7.98	< 0.001
	c50_young	$-2.51x10^{8}$	$1.52x10^{7}$	-16.52	< 0.001
	c50_old	$-2.51x10^{8}$	$1.52x10^{7}$	-16.56	< 0.001
c10_old	c30_young	$-1.75x10^{8}$	$1.52x10^{7}$	-11.54	< 0.001
	c30_old	$-1.66x10^{8}$	$1.52x10^{7}$	-10.94	< 0.001
	c50_young	$-2.97x10^{8}$	$1.52x10^{7}$	-19.51	< 0.001
	c50_old	$-2.97x10^{8}$	$1.52x10^{7}$	-19.55	< 0.001
c30_young	c30_old	$9.00x10^{6}$	$1.52x10^{7}$	0.59	1
	c50_young	$-1.21x10^{8}$	$1.52x10^{7}$	-7.97	< 0.001
	c50_old	$-1.21x10^{8}$	$1.52x10^{7}$	-8.01	< 0.001
c30_old	c50_young	$-1.30x10^{8}$	$1.52x10^{7}$	-8.56	< 0.001
	c50_old	$-1.31x10^{8}$	$1.52x10^{7}$	-8.60	< 0.001
c50_young	c50_old	$-6.00 x10^{6}$	$1.52x10^{7}$	-0.04	1

$SPION_{NIRF-Rh}$: multimodal nanoparticles; BM-MNC: bone marrow mononuclear cells; NIRF: Near-infrared fluorescence; c10: concentration of 10 μg Fe/mL of $SPION_{NIRF-Rh;}$ c30: concentration of 30 μg Fe/mL of $SPION_{NIRF-Rh;}$ c50: concentration of 50 μg Fe/mL of $SPION_{NIRF-Rh}$; CI: confidence interval.

Table S5. ANOVA of the iron quantification internalized into BM-MNC from young and old animals labeled with 10, 30, and 50 μg Fe/mL of $SPION_{NIRF-Rh}$ by ICP-MS.

	Variation source	Sum of squares	Degrees of freedom	Mean square	F	p
**pg Fe/BM-MNC**	Applications	27.73	5	5.55	790.89	< 0.001
	Residual	0.17	24	0.01		
**nº de** $SPION_{NIRF-Rh}$ **/BM-MNC**	Applications	$4.36x10^{9}$	5	$8.75x10^{8}$	810.15	< 0.001
	Residual	$2.59x10^{7}$	24	$1.08x10^{6}$		

$SPION_{NIRF-Rh}$: multimodal nanoparticles; BM-MNC: bone marrow mononuclear cells; ICP-MS: Inductively Coupled Plasma Mass Spectrometry.

Table S6. Post Hoc test of the iron quantification internalized into BM-MNC from young and old animals labeled with 10, 30, and 50 μg Fe/mL of SPIONNIRF-Rh by ICP-MS.

Group	Mean difference	Std. error	t	p

c10_young	c10_old	0.23	0.05	4.277	0.003
	c30_young	-1.31	0.05	-24.73	< .001
	c30_old	-1.11	0.05	-20.95	< .001
	c50_young	-2.33	0.05	-44.07	< .001
	c50_old	-2.10	0.05	-39.75	< .001
c10_old	c30_young	-1.54	0.05	-29.00	< .001
	c30_old	-1.34	0.05	-25.22	< .001
	c50_young	-2.56	0.05	-48.33	< .001
	c50_old	-2.33	0.05	-44.02	< .001
c30_young	c30_old	0.20	0.05	3.77	0.010
	c50_young	-1.02	0.05	-19.33	< .001
	c50_old	-0.79	0.05	-15.02	< .001
c30_old	c50_young	-1.22	0.05	-23.11	< .001
	c50_old	-0.99	0.05	-18.80	< .001
c50_young	c50_old	0.23	0.05	4.31	0.003

$SPION_{NIRF-Rh}$: multimodal nanoparticles; BM-MNC: bone marrow mononuclear cells; ICP-MS: Inductively Coupled Plasma Mass Spectrometry; Std. error: Standard error; c10: concentration of 10 μg Fe/mL of $SPION_{NIRF-Rh;}$ c30: concentration of 30 μg Fe/mL of $SPION_{NIRF-Rh;}$ c50: concentration of 50 μg Fe/mL of $SPION_{NIRF-Rh}$.

Table S7. ANOVA of T2 values of the MRI samples of BM-MNC labeled with $SPION_{NIRF-Rh}$.

Variation source	Sum of squares	Degrees of freedom	Mean square	F	p
Applications	12354.78	3	4118.26	732.52	< 0.001
Residual	67.47	12	5.62		

$SPION_{NIRF-Rh}$: multimodal nanoparticles; BM-MNC: bone marrow mononuclear cells; MRI: Magnetic resonance imaging.

Table S8. Post Hoc test of T2 values of MRI samples of BM-MNC from young and old animals labeled with 50 μg Fe/mL of $SPION_{NIRF-Rh}$.

Group		Mean difference	95% CI		95% CI
			Lower Bound	Upper Bound	
c50_young	ctrl_young	-55.42	1.68	-33.05	< 0.001
	ctrl_old	-56.64	1.68	-33.79	< 0.001
	c50_old	-0.93	1.68	-0.56	1
ctrl_young	ctrl_old	-1.22	1.68	-0.73	1
	c50_old	54.49	1.68	32.50	< 0.001
ctrl_old	c50_old	55.71	1.68	33.29	< 0.001

$SPION_{NIRF-Rh}$: multimodal nanoparticles; BM-MNC: bone marrow mononuclear cells; MRI: Magnetic resonance imaging; ctrl: Control; c50: concentration of 50 μg Fe/mL of $SPION_{NIRF-Rh}$.

Table S9. T test of the iron quantification internalized in BM-MNC from young and old animals labeled with $SPION_{NIRF-Rh}$ by MRI.

	t	Degrees of freedom	p
**pg Fe/BM-MNC**	0.36	6	0.764
**nº of $SPION_{NIRF-Rh}$/BM-MNC**	0.30	6	0.770

$SPION_{NIRF-Rh}$: multimodal nanoparticles; BM-MNC: bone marrow mononuclear cells; MRI: Magnetic resonance imaging; ctrl: Control; c50: concentration of 50 μg Fe/mL of $SPION_{NIRF-Rh}$.
